# Temporal Auditory Resolution, Timed Motor Responses to Auditory Cues, and Phonological Processing in Swedish Children with Cochlear Implants and Normal Hearing Peers

**DOI:** 10.3390/audiolres16050153

**Published:** 2026-10-08

**Authors:** Karolina Löwgren, Birgitta Sahlén

**Affiliations:** 1 Otorhinolaryngology, Department of Clinical Sciences, Skåne University Hospital, Lund University, SE-221 00 Lund, Sweden; 2 Logopedics, Phoniatrics and Audiology, Department of Clinical Sciences, Medical Faculty, Lund University, SE-221 00 Lund, Sweden; birgitta.sahlen@med.lu.se

**Keywords:** children, cochlear implants, gap detection, nonword repetition, reaction time, finger tapping

## Abstract

**Background:** Hearing with a cochlear implant (CI) presents challenges for speech perception, particularly in acoustically complex environments. Auditory temporal resolution, defined as the ability to detect brief changes in an ongoing acoustic signal, is one factor that may contribute to these difficulties. Accurate discrimination of speech sounds is essential for children’s language development and vocabulary acquisition, as subtle temporal variations in speech can alter word meaning. **Methods:** We investigated the ability to detect brief auditory cues at the millisecond level using a gap detection in noise task. Participants also completed non-verbal tasks (reaction time and finger tapping) and a verbal task (nonword repetition), all of which rely on the perception, processing and timing of brief temporal events. Fifteen children with CI were compared with fifteen matched peers with normal hearing. **Results:** We found that the children with CI required gaps approximately twice as long as those required by the children with normal hearing. In contrast, they responded significantly faster to simple auditory stimuli. Both groups timed their motor responses equally well to auditory cues. Lastly, the children with CI were outperformed on correctly repeated nonwords by the children with normal hearing. However, their performance varied across different consonant types, possibly affecting the perceptual clarity of the articulation differently. **Conclusions:** This study explored auditory temporal processing abilities across multiple levels and domains. The findings revealed distinct performance patterns across the different tests. Despite these group differences, the results imply that CIs provide the possibility to perceive brief temporal changes in auditory signals, react fast and time motor responses to auditory cues. The relationships among the different tasks, as well as the extent to which sensitivity to brief acoustic properties contributes to speech processing, require further investigation.

## 1. Introduction

Individuals with cochlear implants (CIs) often experience difficulties related to perception and production of pitch in music, as well as in the perception of certain prosodic features of speech [1,2,3]. In general, CI users appear to perform better in tasks relying primarily on temporal processing, such as maintaining a musical beat [4]. Nevertheless, some CI users exhibit deficits in temporal processing [5], and gaps remain in our understanding of their auditory temporal abilities. Although CI users seem to benefit from relatively coarse temporal cues, they may experience greater difficulty in processing the finer temporal information used in CI signal processing to support pitch perception [6]. Temporal processing is a fundamental aspect of hearing and occurs at multiple levels of the auditory system, ranging from peripheral neuronal sensitivity to cortically integrated processing [7]. It is essential for accurate speech perception and phoneme discrimination [8], where it interacts with spectral transitions and intensity shifts. One component of auditory temporal processing is temporal resolution, defined as the ability to detect brief changes over time in an acoustic signal. Auditory temporal resolution constitutes one of four auditory temporal abilities, together with temporal patterning, temporal integration, and temporal masking [7,9]. Individuals with auditory nerve dys-synchrony or neuropathy have been shown to exhibit deficits in auditory temporal resolution [10,11]. A common way of assessing auditory temporal resolution is to measure the shortest detectable interval between two consecutive sounds, either tones or noise bursts, at which a listener can reliably perceive them as separate events. This measure is commonly referred to as the gap detection threshold. Auditory temporal resolution is believed to develop early in life. Previous research has shown that school-aged children, from approximately seven years of age, can complete gap detection in noise tests without apparent difficulty [12]. To assess the ability to utilize temporal auditory cues and perform temporally precise actions in more integrated tasks, finger tapping synchronized to an external rhythm can be employed. This form of sensorimotor synchronization engages several brain regions involved in temporal processing and motor coordination. Children with CIs have rarely been investigated using this type of non-verbal temporal processing measure. However, atypical finger tapping performance has been reported in children with developmental language disorder and dyslexia, and rhythm production abilities have been linked to language processing skills in developmental language disorder [13,14].

The speech signal contains information that unfolds at multiple temporal rates, including rapid acoustic co-articulatory transitions as short as ~10 milliseconds within and between speech sounds and syllables. Brief temporal variations can alter the meaning of an utterance. One example is voice onset time (VOT), defined as the interval between the release of a stop consonant (plosive), following a silent closure of the vocal tract that blocks the airflow, and the onset of voicing in the subsequent vowel. This duration differs across voiced (/b/, /d/, /g/), voiceless (/p/, /t/, /k/), and aspirated (/p^h^/, /t^h^/, /k^h^/) stop consonants, thereby contributing to their accurate identification and the right meaning of a word. Perception and production of speech sounds can be assessed by the repetition of nonwords, a task in which participants repeat novel phoneme sequences that carry no lexical meaning. The task engages multiple levels of implicit phonological processing, including auditory decoding to phonological working memory, motor planning and production of speech [15]. Poor nonword repetition performance is considered a robust clinical marker of developmental language disorder in children with normal hearing [16,17,18]. Furthermore, children with developmental language disorder have been shown to exhibit difficulties with perception of fast speech [19]. An advantage of nonwords over real words is that performance is less influenced by individual external factors contributing to a child’s word and language acquisition, such as socioeconomic background or a caregiver’s level of education [20]. In everyday listening situations, cognitive top-down processes support speech perception by allowing listeners to use contextual information, expectations, and prior knowledge to compensate for degraded or incomplete auditory input. Such processes are likely particularly important for individuals with CIs, who often rely on contextual cues to facilitate communication in daily life. In nonword repetition tasks, however, these compensatory strategies are largely unavailable because the stimuli lack semantic content and contextual support. Consequently, successful performance depends more directly on the auditory signal and accurate auditory and phonological processing.

Individuals with CIs receive an auditory signal that is both degraded and more variable than that available to listeners with normal hearing. With everyday sound levels fluctuating continuously and background noise often present, listeners naturally face challenges in detecting and interpreting signals that may only be partially audible [9]. This is further compounded by the need to discriminate rapidly changing acoustic and speech cues. Through CIs, such demands may be particularly challenging. Accordingly, counseling of caregivers on how to provide spoken language support with rich and high-quality linguistic input has been highlighted as an important early intervention for children with hearing impairment [21]. A study of experienced adult CI users found no evidence that electrical dynamic range, defined as the difference between minimum and maximum electrode stimulation levels, was associated with better speech recognition outcomes, whereas rehabilitative interventions were positively associated with speech recognition performance [22]. Increased knowledge of the factors contributing to auditory, speech, and language outcomes in children with cochlear implants may facilitate the development of improved device technology and more targeted rehabilitation strategies. Ultimately, spoken language development, effective communication, and participation in society are among the primary goals of pediatric cochlear implantation.

The aim of the present study was to explore the performances across a multilevel range of verbal and non-verbal tasks that depend on the perception and timing of brief auditory events, as well as to compare the results of Swedish children with CIs to age-, sex- and non-verbal IQ-matched children with typical hearing. The research question was: How do children with CIs differ from children with normal hearing in the perception of brief acoustic components and in tasks requiring the use of temporal auditory cues in integrated tasks involving sensorimotor performance and speech processing? To address this question, we examined performance in detecting brief changes in time (gap detection in noise), reacting fast to sound (simple reaction time), producing and keeping a precise tempo by timing motor responses to an auditory rhythm (finger tapping), and repeating novel words of different phonological complexity (nonword repetition). These measures capture different aspects of temporal perception and processing on a millisecond level, while also engaging other auditory and cognitive functions.

## 2. Materials and Methods

### 2.1. Participants

Thirty Swedish-speaking children were included in this study (Table 1). Fifteen of these children (5 males and 10 females) had cochlear implants (CIs) and were, on average, 9.9 years old (*SD* = 1.5). They had received their implants at a relatively early age (mean age at hearing loss diagnosis = 1.6 years *SD* = 0.9, mean age at first implantation = 2.2 years *SD* = 1.0) and were recruited during two summer camps held in southern Sweden in 2013 and 2017, organized by Barnplantorna (a Swedish interest organization for children with CIs or hearing aids and their families, https://barnplantorna.se/, accessed on 10 July 2026). All attended integrated schools with typically hearing Swedish-speaking classmates. The remaining fifteen children (5 males and 10 females) had normal hearing (NH) and were, on average, 9.6 years old (*SD* = 1.1). They were matched to the CI participants on age, sex and non-verbal intelligence quotient (IQ). These participants were selected from a larger sample of typically developing children with NH who had previously been assessed using the same test procedures and equipment [23,24]. In conjunction with the testing sessions, caregivers completed questionnaires regarding hearing etiology, language background, handedness, participation in rhythm-related activities involving auditory and motor skills (e.g., playing a musical instrument or dancing), and parental educational level. No history of speech and language therapy was reported for any of the children in the NH group.

### 2.2. Procedure

Gap detection in noise (GIN), reaction time (RT), finger tapping, and nonword repetition (NWR) were administered to assess the ability to perceive brief acoustic stimuli and to coordinate speech articulation and non-verbal motor responses to auditory input. Raven’s Colored Progressive Matrices were also administered during the session to assess non-verbal reasoning (IQ), which served as one of the matching criteria between the CI and NH groups [25]. In addition, participants in the NH group underwent a standard hearing screening using pure-tone audiometry at 125–8000 Hz at 20 dB HL (GSI 66 screening audiometer, TDH-39 headphones, modified Hughson–Westlake method in accordance with ISO 8253-1) [26]. All tests were conducted binaurally in the participants’ best aided listening condition for the CI users (bilateral CI = n13, bimodal hearing = n2) and presented in a sound field via a loudspeaker. An exception was the GIN test, which was administered monaurally through headphones to NH participants, with the right ear tested first. All sound sources and presentation levels were calibrated and verified at the test distance using a calibrator and sound level meter (4230 Brüel & Kjær, Nærum, Denmark/DSP80 Larson & Davis Type 1, Provo, UT, USA/ISO-TECH SLM-52N, Corby, UK). Prior to each subtest, participants were asked to rate the presentation level to ensure that all stimuli were clearly audible, comfortable, and neither too weak nor too loud.

**Table 1:** Background factors. For the 15 normal hearing children (NH group) and the 15 children with cochlear implants (CI group).

	NH Group	CI Group
Participants, *n*	15	15
Age at test in years, mean (*SD*)	9.6 (1.1)	9.9 (1.5)
IQ standard score, mean (*SD*)	108 (14)	107 (13)
IQ percentile, mean (*SD*)	65 (29)	63 (26)
Right ear 4fPTA ^a^ in dB HL, mean (*SD*)	20.6 (1.1)	-
Left ear 4fPTA ^a^ in dB HL, mean (*SD*)	20.5 (1.1)	-
Hearing problems, *n*	0	-
History of speech/language therapy, *n*	0	-
Bilateral cochlear implants, *n*	-	13
Contralateral hearing aid, *n*	-	2
Known hearing etiology, *n*	-	6 ^b^
Age at hearing loss diagnosis in years, mean (*SD*)	-	1.6 (0.9)
First ear implantation age in years, mean (*SD*)	-	2.2 (1.0)
Second ear implantation age in years, mean (*SD*)	-	2.8 (2.1)
Bilingual, *n*	4 ^c^	4 ^d^
Parent’s education	^e^	^f^
Musical instrument practice, *n*	2 ^g^	7 ^h^
Dance classes, *n*	-	8 ^i^
TV/Computer gaming, *n*	-	14 ^j^

Comments: ^a^ Pure tone threshold average in hearing level decibel (dB HL) calculated on four frequencies (500, 1000, 2000 and 4000 Hz), where typically ≥25 dB HL is considered hearing impairment [27]. ^b^ 2 Hereditary, 2 LVAS, 1 Usher, 1 Meningitis ^c^ 2 Danish, 1 English, 1 Arabic ^d^ 1 Danish, 1 English, 1 Spanish, 1 Bosnian ^e^ NH children recruited from elementary schools where 68% of the pupils had at least one parent with higher education in the year of the data collection (retrieved from the Swedish National Agency for Education’s statistics about preschools and schools). ^f^ Lowest educational level in the families: (1) Compulsory school = 0%. (2) Upper Secondary school = 42%. (3) Higher education diploma/Bachelor’s degree/Master’s degree/Doctoral degree = 58%. (4) Other = 0%. ^g^ Piano ^h^ Violin, piano, recorder, saxophone, clarinet. ^i^ Last 2–6 years. ^j^ 0.5–20 h/week.

#### 2.2.1. Gap Detection in Noise Test

To assess auditory temporal resolution, gap detection was measured using a psychoacoustic Gaps-In-Noise test (GIN) implemented in the portable computer audiometer Software for Mobile Auditory Physiology (version SMAPH28; developed by Jan Grenner, Lund, Sweden) [28]. Participants were presented with silent gaps ranging from 1 to 20 ms embedded in white noise centered at 1000 Hz. For children with CIs, stimuli were presented in a sound field at 65 dB SPL at a distance of 1 m from a loudspeaker (SPA11, Fostex, Akishima, Tokyo, Japan/Companion 2 series III, Bose Corporation, Framingham, MA, USA). For children with NH, stimuli were presented monaurally through Sennheiser HDA-200 headphones at 55 dB SPL, with each ear tested separately. The test consisted of 30 trials. Participants reported whether they perceived each stimulus as one continuous noise or as two separate noise segments. The gap detection threshold, defined as the shortest detectable gap duration, was estimated using an adaptive RMLSP algorithm [29]. For the NH group, the mean threshold across the right and left ears was calculated and used in the subsequent statistical analyses.

#### 2.2.2. Reaction Time Test and Isochronous Interval Finger Tapping

To assess the ability to produce timed motor responses to auditory stimuli, simple reaction time (RT) and paced isochronous interval timing were studied though finger tapping [30]. The stimulus during the paced finger tapping task consisted of an isochronous 30 ms transient click repeated 15 times at 524 ms intervals and presented at 65–70 dB(A), corresponding to each participant’s most comfortable listening level, from a loudspeaker connected to a Microtower PC (HP Compaq dx2000, Hewlett-Packard Company, Palo Alto, CA, USA) positioned 0.5 m from the participant. Participants were instructed to synchronize their taps to the rhythm of the clicks and to maintain the same rate when the auditory stimulus ceased. They then continued tapping until they were instructed to stop after 70 taps. As performance on finger tapping tasks may improve with practice, a shorter training (15 synchronization taps and 31 continuation taps) was administered prior to the four test trials. Both the synchronization phase and the self-paced continuation (production phase) were analyzed in terms of inter-tap intervals (inter-response intervals, IRIs) and timing variability. During the RT task, participants responded to one single transient as quickly as possible. Following 12 practice trials, 30 test trials were administered. The stimulus was presented at random intervals within a 2300–4000 ms time window. In both tasks, participants used the index finger of their dominant hand to tap the space bar while keeping their hand palm-down. Mean, median and standard deviation values for response times and inter-tap intervals were calculated and used in the statistical analyses.

#### 2.2.3. Nonword Repetition Test

Implicit phonological processing was assessed using a nonword repetition (NWR) task comprising 24 pseudowords consisting of three or four syllables and lacking any real-word equivalents in Swedish. The nonwords were prerecorded by a female speaker with a central Swedish accent using a computer program (SIPS) [31], developed from Sahlén et al. [32], and were presented at 65–70 dB(C) from a loudspeaker positioned 0.5 m from the participant. Participants were instructed to listen to the nonwords and repeat them exactly as heard immediately after each presentation. The session was videorecorded for transcription purposes. In total, the material contained 84 vowels and 120 consonants. When grouped according to manner of articulation (i.e., how airflow is shaped or restricted within the vocal tract to produce speech sounds), the consonants comprised 47 plosives (/p/, /t/, /k/, /b/, /d/, and /g/), 23 fricatives (/f/, /s/, /h/, /v/, and /j/), 21 nasals (/m/, /n/, and /ng/), 20 laterals (/L/), and 9 /r/-sounds. The degree of phonological complexity varied across the test material, which was divided into three subtests. The first subtest contained 8 words without any consonant clusters (e.g., /’ka:simum/ and /nesso’lå:/). The second subtest contained 8 words with consonant clusters that are allowed according to Swedish phonotactic rules (e.g., /’spja:bitte/ and /muje’stra:l/). The third subtest contained 8 words with consonant clusters that violate Swedish phonotactics constraints (e.g., /‘tke:nema/ and /seka’la:gb/). Following transcription, the percentage of correctly repeated complete nonwords, vowels, and consonants was scored both for the task as a whole and separately for each subtest. In addition, consonant accuracy was analyzed separately for the five consonant types. Minor articulatory variations were accepted. For the binary scoring of whole nonwords as correct or incorrect, phoneme substitutions, omissions, additions, and incorrect stress placement were scored as errors. In the vowel and consonants scoring, phoneme substitutions and omissions resulted in a deduction of one point from the total score, whereas phoneme additions did not affect the score. The reversal of two phonemes, provided that both phonemes were produced, resulted in a deduction of one point. Two trained speech and language pathologists or researchers independently scored the material. Inter-rater reliability was 97% regarding the NH participants’ scores and 99% for the CI participants’ scores.

### 2.3. Statistics

According to the Shapiro–Wilk test of normality, which is appropriate for sample sizes below 50 participants, not all variables were normally distributed within the two study groups. Therefore, non-parametric statistical tests were used for all analyses.

Comparisons between the CI and NH groups for GIN thresholds, reaction time (RT), and finger tapping performance were conducted using the Mann–Whitney U test. Since seven Mann–Whitney U tests were performed, Bonferroni correction was applied to control for multiple comparisons, resulting in an adjusted significance level of *p* < 0.007. Effect sizes were calculated and interpreted according to Cohen’s conventions for *r* (0.1 = small, 0.3 = medium, and 0.5 = large).

The Kruskal–Wallis H test (one-way ANOVA on ranks) was used to compare the multiple nonword repetition (NWR) outcome measures, both for the total scores and for the different subtests, between groups. Because twelve Kruskal–Wallis tests were conducted, Bonferroni correction was applied to adjust for multiple comparisons, resulting in an adjusted significance level of *p* < 0.004. Effect sizes were calculated and interpreted according to epsilon-square (*ε*^2^) classification (0.01 = small, 0.08 = medium, 0.26 = large). The results for the five specific consonant types in the NWR task were analyzed only for the total test scores, as the number of observations was considered too small for separate analyses of the three subtests. Bonferroni correction was applied for these five comparisons resulting in an adjusted significance level of *p* < 0.01.

Within-group comparisons of NWR performance across subtests and consonant types were conducted using the Friedman test. Bonferroni correction for these four comparisons resulted in an adjusted significance level of *p* < 0.0125. Effect sizes were calculated using Kendall’s coefficient of concordance (Kendall’s *W*) and interpreted according to commonly used conventions (0.1 = small, 0.3 = medium, and 0.5 = large). When significant effects were identified, post hoc pairwise comparisons were performed using the Wilcoxon signed-rank test. Effect sizes were calculated using *r* and interpreted according to Cohen’s conventions. Bonferroni correction of these multiple post hoc comparisons resulted in an adjusted significance level of *p* < 0.002.

For the principal effect size estimates, 95% bootstrap confidence intervals (5000 resamples) were calculated for the main outcome measures.

## 3. Results

### Differences in Gap Detection Thresholds, Reaction Times, and Nonword Repetition, but Not in Finger Tapping, Between the Groups

**Figure 1:**
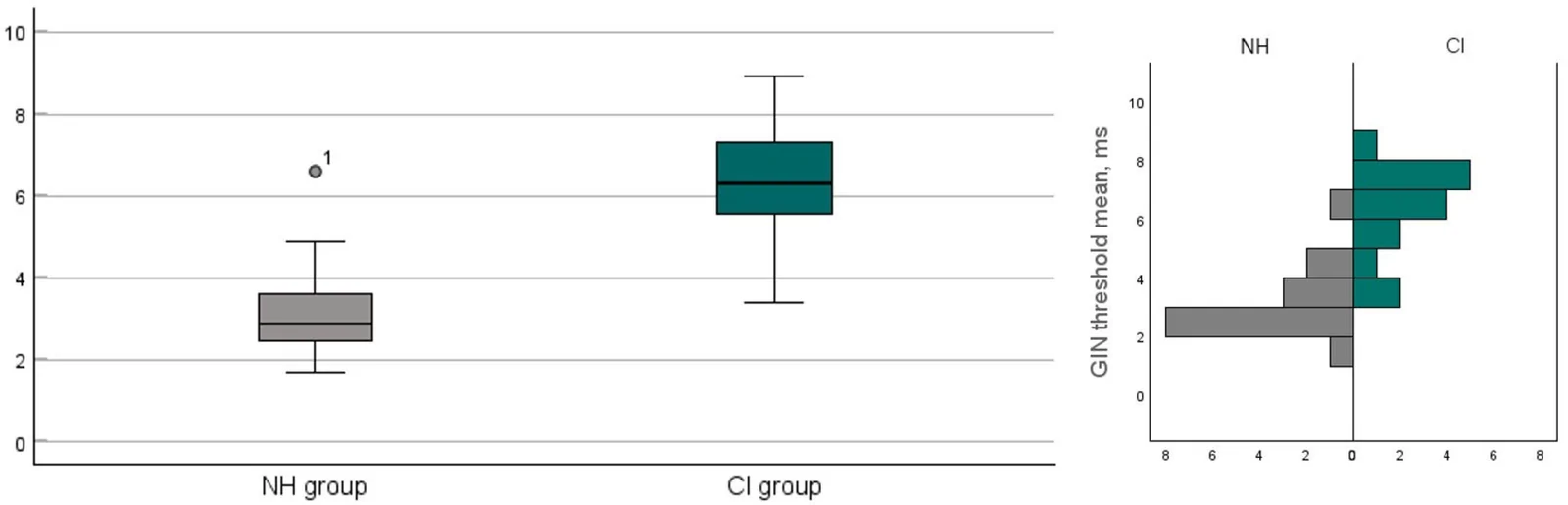
Gap detection in noise (GIN) thresholds in milliseconds (ms) for the 15 children with normal hearing (NH) and the 15 children with cochlear implants (CIs). The left panel displays boxplots of mean GIN thresholds for each group, while the right panel shows the corresponding frequency distributions. Mean GIN thresholds were approximately twice as long in the CI group as in the NH group. The numbered point (1) in the NH group represents an outlier.

The children with cochlear implants (CIs) exhibited higher gap detection thresholds in the gap detection in noise (GIN) task (mean = 6.3, *SD* = 1.5) than the children with normal hearing (NH) (mean = 3.2, *SD* = 1.3), requiring nearly twice the gap duration to detect a silent interval within the noise (Figure 1). An independent-samples Mann–Whitney U test indicated a significant difference between the groups (*U* = 209.00, *Z* = 4.004, *p* < 0.001), with a large effect size (*r* = 0.731). The corresponding rank-biserial correlation was 0.858 (95% bootstrap confidence interval [0.636, 1.000]). Statistical significance for Mann–Whitney U tests was evaluated using a Bonferroni-adjusted threshold of *p* < 0.007. Within the NH group, which was tested monaurally, no significant difference was found between right-ear (mean = 2.7, *SD* = 1.0) and left-ear (mean = 3.6, *SD* = 2.1) GIN thresholds according to a Wilcoxon signed-rank test (*Z* = −1.647, *p* = 0.100).

**Figure 2:**
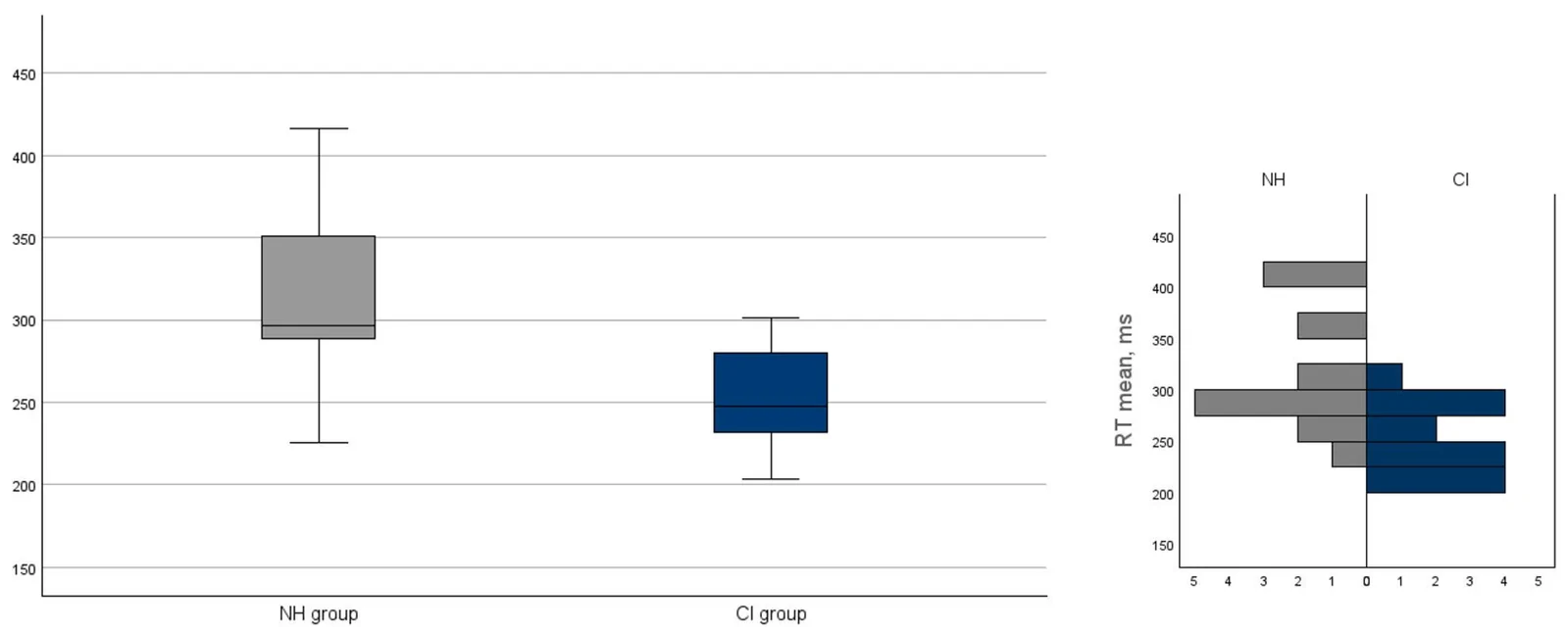
Reaction times (RTs) in milliseconds (ms) for the 15 children with normal hearing (NH) and the 15 children with cochlear implants (CIs). The left panel displays boxplots of mean RT for each group, while the right panel shows the corresponding frequency distributions. Mean RTs were shorter (faster) in the CI group than in the NH group.

The children in the CI group demonstrated significantly faster reaction times (RTs) (mean = 254, *SD* = 30 and median = 250, *SD* = 30) than the children in the NH group (mean = 316, *SD* = 59 and median = 314, *SD* = 61) (Figure 2). Significant group differences were observed for both mean RT (*U* = 35.00, *Z* = −3.215, *p* = 0.001, *r* = 0.587) and median RT (*U* = 37.00, *Z* = −3.132, *p* = 0.002, *r* = 0.572). For the primary comparison, mean RT, the corresponding rank-biserial correlation was −0.689 (95% bootstrap confidence interval [−0.929, −0.360]). These differences remained significant when evaluated against the Bonferroni-adjusted significance threshold of *p* < 0.007 and were associated with large effect sizes according to Cohen’s conventions.

**Figure 3:**
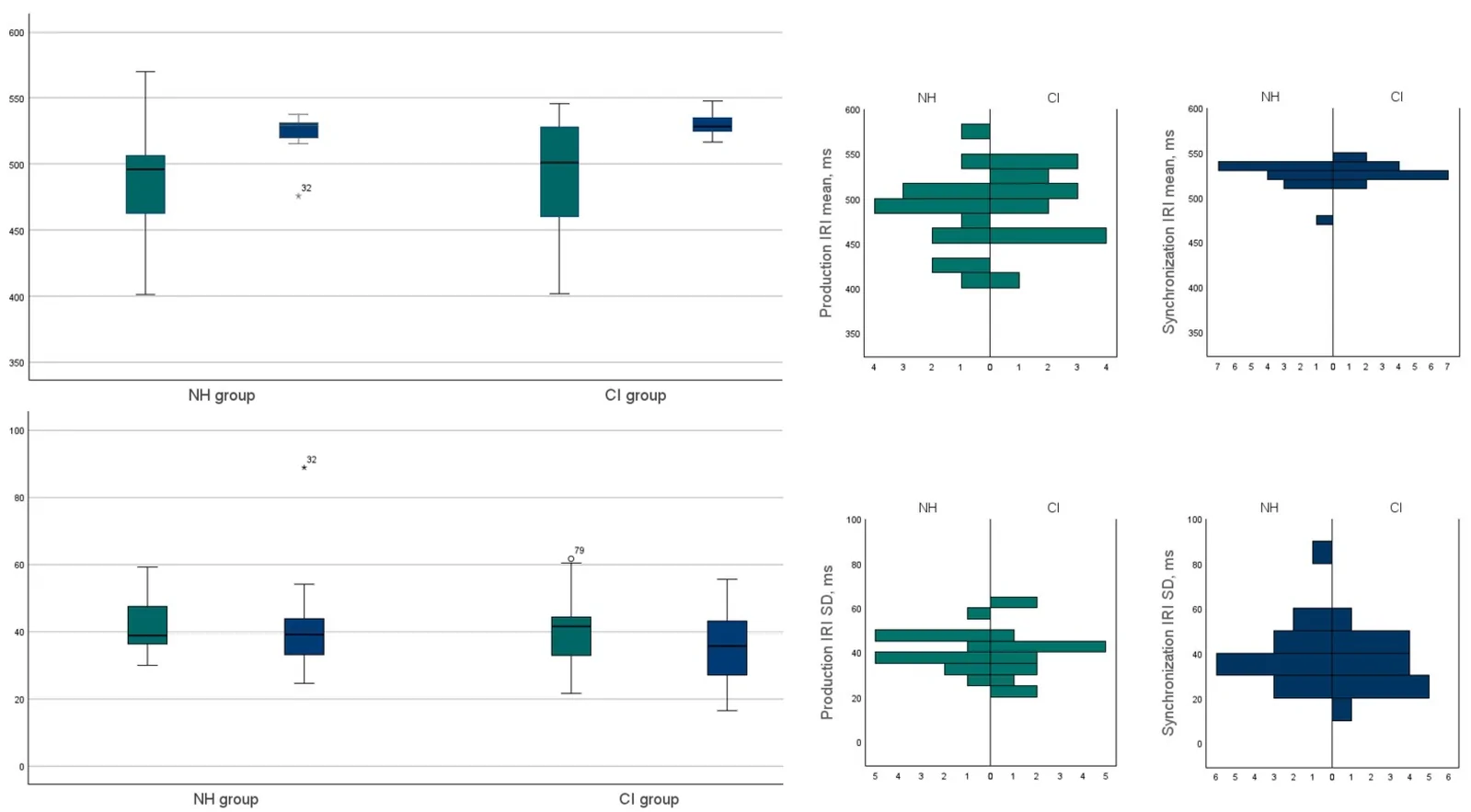
Finger tapping inter-response intervals (IRIs) and timing variabilities in milliseconds (ms) for the 15 children with normal hearing (NH) and the 15 children with cochlear implants (CIs). Similar performance patterns were observed in the two groups. The left panels display boxplots of mean IRI (**upper panel**) and timing variability, expressed as the standard deviation (SD) of the IRI (**lower panel**), during both the synchronization phase (dark blue) and the self-paced production phase (green). The numbered point and asterisks indicate outliers. The corresponding frequency distributions for each measure and group are shown in the panels to the right.

In contrast, the CI and NH groups performed similarly on the finger tapping task (Figure 3). No significant group differences were found in mean tapping tempo, expressed as inter-response interval (IRI), either during the synchronization phase to the isochronous 524 ms metronome interval (*U* = 135.00, *Z* = 0.933, *p* = 0.351, *r* = 0.170) or during the following self-paced continuation (production phase) without external auditory pacing (*U* = 127.00, *Z* = 0.601, *p* = 0.548, *r* = 0.110). Effect sizes for both measures were small. Mean IRI during the synchronization phase was 530 ms (*SD* = 9) in the CI group and 524 ms (*SD* = 15) in the NH group. During the production phase, mean IRI was 493 ms (*SD* = 42) in the CI group and 485 ms (*SD* = 44) in the NH group. Further, no significant group differences were observed in tapping variability, measured as the standard deviation of the IRI, either during the synchronization phase (*U*= 88.00, *Z* = −1.016, *p* = 0.310, *r* = 0.186) or during the production phase (*U* = 97.00, *Z* = −0.643, *p* = 0.520, *r* = 0.117). Effect sizes were small for both measures. Mean IRI variability in the CI group was 35 ms (*SD* = 11) during the synchronization phase and 40 ms (*SD* = 12) during the production phase, compared with 41 ms (*SD* = 16) and 42 ms (*SD* = 8), respectively, in the NH group.

**Figure 4:**
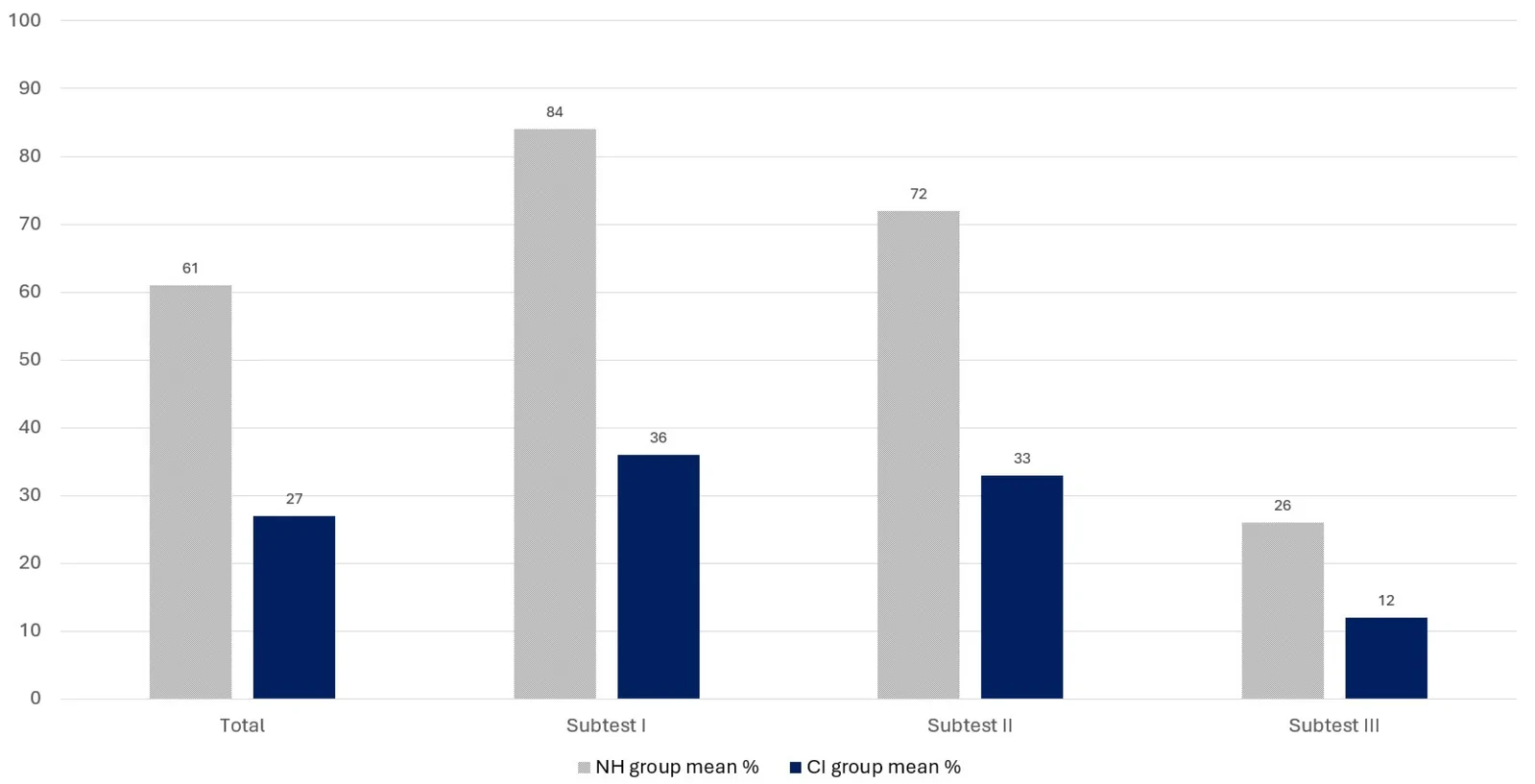
Mean percentage correctly repeated complete nonwords for the 15 children with normal hearing (NH) and the 15 children with cochlear implants (CIs). The NH group (gray bars) repeated a higher percentage of nonwords correctly than the CI group (dark blue bars), both overall and in each of the three subtests.

On the nonword repetition (NWR) task, the children in the NH group outperformed the children in the CI group on both the total percentage of correctly repeated whole nonwords and the first two subtests analyzed separately, as confirmed by Kruskal–Wallis H tests (one-way ANOVA on ranks), with medium-to-large effect sizes (*ε*^2^) (Figure 4, Table 2). For the overall NWR score, the group difference was associated with a large effect size (*ε*^2^ = 0.737, 95% bootstrap confidence interval [0.709, 0.754]). Similar results were observed for the percentage of correctly repeated vowels and consonants analyzed separately, where the NH group achieving higher scores than the CI group both overall and in the first two subtests. These differences remained significant when evaluated against the Bonferroni-adjusted significance threshold of *p* < 0.004.

**Figure 5:**
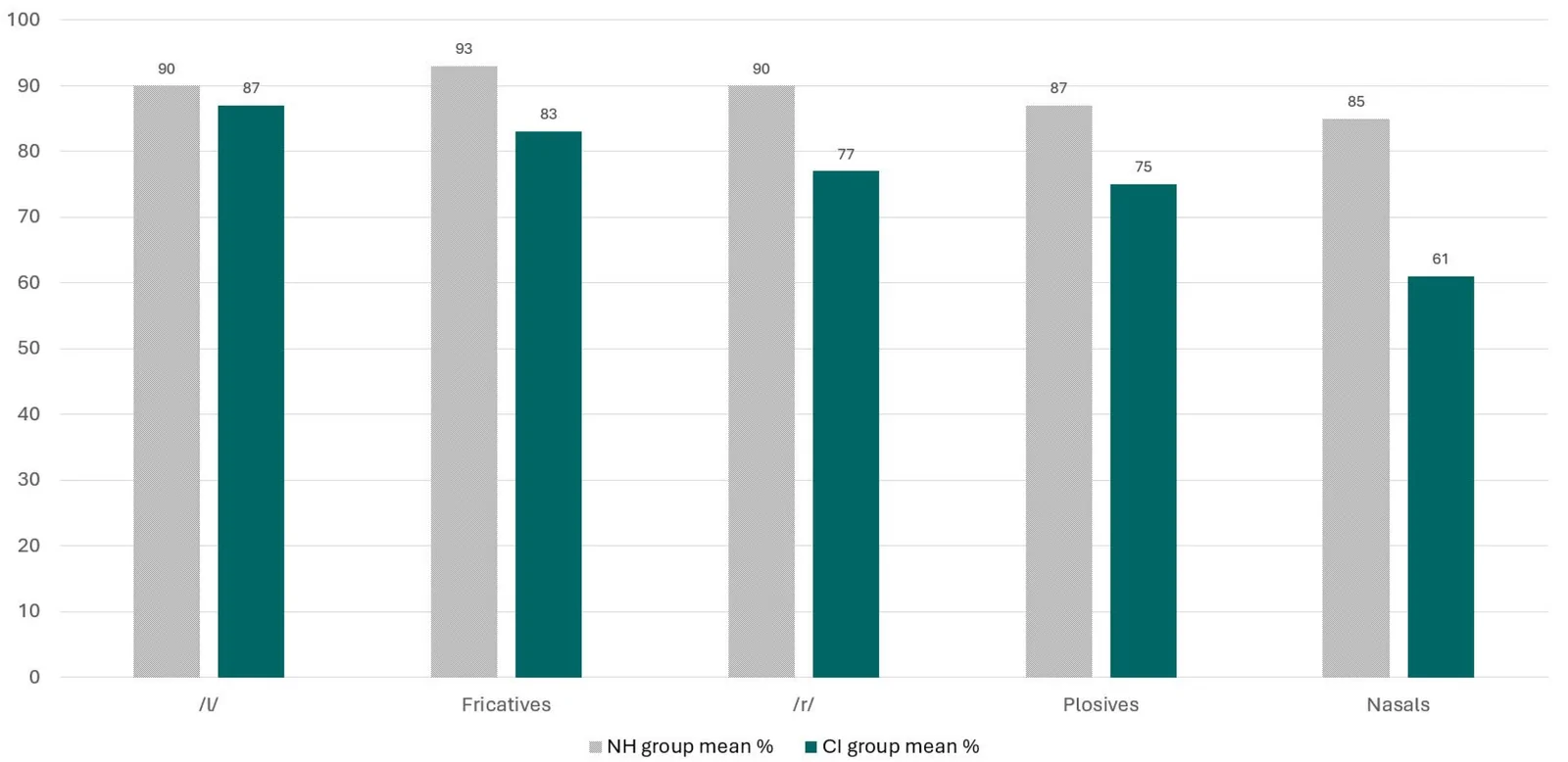
Mean percentage of correctly repeated consonant types during nonword repetition (NWR) for the 15 children with normal hearing (NH) and the 15 children with cochlear implants (CIs). Results are presented for five consonant types based on manner of articulation: laterals (/L/), fricatives, /r/ sounds, plosives, and nasals. The NH group (gray bars) correctly repeated a significantly higher percentage of consonants than the CI group (green bars) across all consonant types except laterals (/L/), for which no significant group difference was observed.

Furthermore, the NH group correctly repeated significantly more consonants across the specific consonant types of plosives, fricatives, /r/, and nasals than the CI group. In contrast, no significant group difference was observed for /L/ sounds (laterals) (*p* = 0.314), which the children with CIs repeated almost as accurately as the children with NH (Figure 5, Table 3). All group differences remained significant when evaluated against the Bonferroni-adjusted significance threshold of *p* < 0.01 and were associated with large effect sizes (*ε*^2^). The largest difference between the groups was observed for nasals, which appeared to be the most difficult consonant type for children with CIs to repeat.

An increase in difficulty across the three subtests, particularly in the third subtest containing consonant clusters that do not occur in Swedish, was observed in both groups. Significant within-group differences in consonant repetition scores were found between the first two subtests and the third subtest, as revealed by Friedman tests and post hoc Wilcoxon signed-rank tests with Bonferroni correction for multiple comparisons (significance level adjusted to *p* < 0.0125) (Table 4). Effect sizes (Kendall’s *W*) ranged from medium to large. Significant differences in consonant type scores were also observed in both groups. Post hoc Wilcoxon signed-rank tests with Bonferroni correction for multiple comparisons (significance level adjusted to *p* < 0.002) revealed several significant pairwise differences among consonant types for only the CI participants, with large effect sizes (*r*). There were also small to large effect sizes for non-significant post hoc comparisons within both groups.

## 4. Discussion

### 4.1. Distinct Performance Patterns Across Tests

In this explorative study, we compared the performance of Swedish children with cochlear implants (CIs) and matched children with normal hearing (NH) across a range of tasks involving the perception and processing of brief auditory events. The findings revealed distinct performance patterns across the different tests. First, our children with CIs demonstrated poorer auditory temporal resolution than our children with NH, measured by detecting gaps in noise (GIN). Within the present test setup, the CI group required gap durations that were approximately twice as long as those required by the NH group to be able to detect a gap within the noise. Second, and in contrast, the CI group exhibited significantly faster reaction times (RTs) to brief auditory stimuli than the NH group. Third, no significant group differences were evident in our sample regarding sensorimotor timing of responses to auditory external and internal feedback, measured by keeping an isochronous rhythm through finger tapping, either in inter-tap intervals (tapping tempo) or in tapping variability. Fourth, the nonword repetition (NWR) scores revealed more pronounced phonological processing difficulties in the CI group than in their NH peers. It also showed varying level of performance across different types of consonant phonemes in the CI group particularly, indicating that certain phonological contrasts may pose greater challenges than others.

#### 4.1.1. Finger Tapping and Reaction Times

With our small sample size and the analyses conducted, we cannot rule out the possibility that differences in finger tapping performance between children with CI and NH may emerge in a larger sample. The two groups performed similarly with respect to both inter-response intervals and tapping variability, suggesting a broadly comparable ability to maintain a regular rhythm using auditory feedback. Nevertheless, small effect sizes were observed for the finger tapping measures, although these findings are difficult to interpret. The CI group showed slightly better timing because of the lower variability during both test phases and performed closer to the intended tempo during the self-paced continuation (production phase). In contrast, the NH group tapped closer to the intended tempo during the synchronization phase. Despite these subtle differences, the overall pattern of results indicated comparable performance between these two groups across all finger tapping measures. Our findings are consistent with previous studies showing that adult CI users perform on par with NH non-musicians regarding measures of rhythm perception and tapping synchronization [33,34,35]. The synchronization performance observed in our sample also appears comparable to that reported for a large sample of 12-year-old typically hearing children assessed using a similar experimental paradigm [36]. Our data indicate that even with a degraded hearing function, sensorimotor timing might be preserved in our CI group, unlike children with developmental language disorder, who often show impaired rhythm tapping [13,14]. Finger tapping is not only a measure of sensorimotor timing but also places demand on attention, concentration, and motor coordination. Although the task is considered a mainly explicit conscious task, it also involves subconscious implicit mechanisms [37]. The two phases of the task engage partly different timing processes. During the synchronization phase, participants align their taps with an externally presented isochronous metronome rhythm. During the continuation phase, they maintain the same rhythm without external auditory input. This latter self-paced phase may place greater demands on internal timing representations and other cognitive resources, potentially contributing to the larger interindividual variability often observed. Finger tapping performance also matures during childhood in individuals with NH [38]. It is therefore also likely that children with CIs similarly develop and refine these sensorimotor timing abilities as the central nervous system matures and motor functions and coordination improve. Further research is needed to clarify how children with CIs perform on tasks involving auditory-guided motor timing across childhood, adolescence, and adulthood in comparison with their peers with NH.

The finding of faster RTs in the CI group was unexpected, given the reduced auditory input typically associated with cochlear implantation. Previous studies have reported longer RTs in adult CI users than in NH adults when performing more complex tasks, particularly under challenging listening conditions [39]. Increased listening effort in such situations may negatively affect processing speed. In the present study, our NH children showed RT comparable to those previously reported for a larger sample of NH children (n42, RT median = 328 ms), whereas our CI group, despite responding faster than their NH peers, did not reach the RT levels reported for NH adults tested under similar conditions (n44, RT median = 191 ms) [23]. The reasons underlying the faster RTs observed in the CI group remain unclear. The finding may represent a chance result related to the limited sample size and should therefore be interpreted with caution until replicated in larger studies. We do not know if it is possible that characteristics of auditory processing through a CI contributed to the observed pattern. CI hearing differs fundamentally from typical hearing in terms of the acoustic, mechanical, and electrical transmission of auditory information. Rather than relying on normal cochlear transduction, intracochlear electrodes directly stimulate auditory nerve fibers and generate a distinctive pattern of neural activation and synchronization. This may also have led to differences in perceived loudness or stimulus salience between our two groups. Furthermore, participation strategies, task-specific factors, or other potential group differences may have contributed to the observed performance.

#### 4.1.2. Gap Detection in Noise Thresholds

Previous studies have demonstrated significantly elevated GIN thresholds in adults with CIs compared with NH controls, with implanted adults exhibiting even higher thresholds than the children with CIs in the present study [40,41], which seems to be consistent with typically better hearing performance in early implanted children compared to adult recipients. Furthermore, previous research has reported positive correlations between gap detection thresholds and implantation age in children, indicating that children implanted at younger ages are able to detect shorter gaps [5]. It should be noted that the data collection for the present study was conducted 2013 and 2017. Consequently, the participating children were typically implanted at older ages than is common in current clinical practice. It therefore remains unclear whether the observed group differences would be reduced in cohorts implanted at younger ages or in a larger sample. Although a contemporary CI is capable of transmitting temporal information with high precision, several characteristics of CI-mediated hearing may contribute to the poorer temporal resolution observed in the CI group. Auditory temporal resolution also depends partly on the cochlear function [9], which is absent in CI users and only partially replicated through a CI. In addition, performance on gap detection tasks is influenced by methodological factors, including stimulus level. For auditory stimuli with durations of up to a couple of hundred milliseconds, perceived loudness is affected by temporal integration [42]. Consequently, shorter stimuli generally require higher presentation levels than longer stimuli to achieve equivalent detectability, as the energy accumulates over time within the auditory system [9]. An association between loudness and pure tone gap detection thresholds has previously been demonstrated in CI users [43]. In NH children, GIN thresholds are typically reported to be approximately 4–5 ms when stimuli are presented at 50 dB sensation level (SL) [12,44,45], although threshold estimates vary depending on stimulus characteristics and testing methodology [46]. In addition, binaural listening results in loudness summation, whereby hearing thresholds are reduced by a few decibels compared with monaural listening due to the integration of input from both ears. In the present study, the presentation level was 10 dB higher in the CI group (65 dB SPL, corresponding approximately to the level of normal conversational speech at a distance of one meter). Furthermore, all CI users had access to binaural hearing when completing the test through a loudspeaker, whereas participants with NH were tested monaurally using headphones. Depending on the characteristics of the CI processor and stimulation strategy, pure-tone thresholds in CI users may be elevated by approximately 5–10 dB relative to the conventional upper limit of normal hearing (20–25 dB HL across frequencies up to 8000 Hz). Given these factors and the considerable interindividual variability among CI users, it may have been preferable to present stimuli at a sensation level (dB SL) or at a predefined level above each participant’s pure-tone average. Consequently, we cannot exclude the possibility that some of the observed group differences were influenced by the differing test conditions. All participants were asked about the sound level and reported that they could hear the stimuli clearly. Although binaural hearing is generally considered advantageous [47,48], it remains unclear whether the bilateral CIs (or bimodal configurations used by two participants) may have introduced interaural synchronization asymmetries that affected performance [49]. In NH children, including those in the present study, GIN thresholds generally do not differ between the right and left ears, and no substantial maturational asymmetries between the ears have been reported from school age through adolescence [12,44].

#### 4.1.3. Nonword Repetition Performance

Our CI children made significantly more errors than their NH peers when repeating nonwords, both regarding complete nonwords accuracy and correct repetition of vowels and consonants in total and in the first two subtests. Consistent with our findings, previous studies have generally reported superior nonword repetition performance in NH children compared to same-age CI children [31,50], although scoring procedures may vary somewhat across studies. The NWR performance of our NH group was comparable to that reported for NH children of similar ages in previous research [31]. Earlier studies of Swedish-speaking children with CIs that employed the same or similar NWR tasks reported lower levels of performance than those observed in our CI group [50,51]. This discrepancy may be attributable to differences in participant characteristics, including younger age and shorter duration of CI experience in those previous cohorts. The scores usually improve with age and may also be influenced by implantation age as well as other individual and rehabilitative factors. Overall, our results support previous findings suggesting that NWR remains particularly challenging for children listening through CI hearing. Although many children with CIs can function close to normal, some difficulties in hearing and perceiving speech may persist throughout life, particularly in acoustically demanding listening situations involving background noise [3,52]. The increased difficulty observed in the third subtest for both groups, together with a more pronounced within-group decline in performance among the NH children relative to the preceding subtests, suggests that the NH participants were particularly sensitive to phoneme combinations that violate Swedish phonotactic constraints. This finding also provides an indication of the level of difficulty that the CI recipients already showed during the earlier subtests, which contained either no consonant clusters (first subtest) or familiar consonant clusters (second subtest). Importantly, the nonwords were presented as prerecorded auditory stimuli without any accompanying visual speech information, which pose challenges for CI users, who often benefit from redundant cues from different modalities, when interpreting spoken language.

Concerning consonant phoneme types classified by manner of articulation, our study revealed significant differences between the two groups for all consonant types except laterals (/l/-sounds), which also appeared to be the easiest consonant sounds for these children with CIs to repeat. Repetition of nasals (/m/, /n/, and /ng/) resulted in the greatest difference between the groups and the lowest percentage of correct repetitions within the CI group. Both laterals and nasals are voiced consonant types, but they have weaker spectral energy than vowels. Nasals may be more difficult to distinguish from the ongoing speech signal. Their characteristic nasal resonance can easily be masked by surrounding sounds, making them potentially less distinct for CI users. Consonants are generally short in duration and are therefore often more difficult to detect than vowels [9]. The within-group differences observed between consonant types in our study indicate that the level of difficulty in perceiving and repeating nonwords varies depending on phoneme type, particularly in children with CIs. Plosives, which are characterized by distinct temporal cues, including a blockage of airflow before release at the place of articulation and differences in voice onset time (VOT) between voiced and voiceless variants, yielded the second-lowest scores after nasals in the CI group. Speech sounds containing a substantial proportion of their intensity in the higher frequencies range, such as fricatives, like /s/ and /f/, are often described as distinct and easy to perceive through a CI among Swedish-speaking individuals. This pattern was also reflected in our results. No significant difference was found between the two consonant types with the highest performance, laterals and fricatives. When considering place of articulation rather than manner of articulation, coronal consonants (produced with the front part of tongue against or approaching the roof of the mouth), such as /l/ and /s/, have been found to be easier for children with CIs to accurately reproduce in NWR. In contrast, they tend to struggle more with labials consonants (involving lip closure), such as /m/ and /b/, and dorsal consonants (produced with the back of the tongue against or approaching the roof of the mouth), such as /ng/ and /g/ [53]. During NWR, perception of short temporal intervals and brief acoustic transitions represents only one aspect in processing the ongoing speech signal. The specific contribution of temporal processing to the results obtained in our study remains unclear. Perception of frequency and intensity changes, together with other speech perception and speech production processes, also contribute to performance.

Children with CIs often exhibit delayed language development prior implantation as a consequence of limited auditory access caused by their hearing loss. Following implantation, spoken language development continues and additional language exposure and support may be required for these children to catch up with and achieve language skills comparable to those of their NH peers. In the present study, the CI and NH children were matched by chronological age. Despite the considerable time elapsed since first implantation at the time of testing (mean = 7.8 years, *SD* = 1.7, span 4.8–11.3), it is unclear whether the CI group had yet attained language abilities comparable to those of their NH peers. Consequently, comparisons of NWR performance between children with CI and younger NH children may, in some respects, be more appropriate. Nevertheless, NH children aged 5–6 years have been reported to achieve higher rates of correct consonant repetition in NWR tasks [31] than most participants in the present CI group, despite all CI participants being at least 8 years of age.

### 4.2. Limitations

All tasks included in the present study involve some degree of auditory temporal perception and processing. However, the study was not designed to determine whether, or to what extent, the different measures overlap or reflect any common underlying mechanisms. This question warrants further investigation in studies specifically designed to examine the relationships between these abilities. Moreover, performance on these tasks depends on a range of processes beyond temporal processing alone. Speech and sound perception rely on the integration of temporal, spectral, and intensity-related cues, all of which contribute to successful auditory perception. Further investigation of speech perception in a broader linguistic context, including analysis of surrounding phonemes, lexical context, and phoneme confusion patterns, could provide valuable insights into perceptual processing in CI hearing. Such approaches may contribute to a more comprehensive understanding of how temporal and spectral cues interact in this population. Cognitive processes such as concentration, motivation, listening effort, motor control, and other task-specific response demands may also influence performance in tasks such as those used in our study. Successful task completion depends not only on perceptual abilities but also on the allocation of cognitive resources to meet task demands [54]. The measures are also sensitive to methodological factors. Comparisons between individuals with normal hearing and those with hearing impairment can be challenging, as differences in audibility may influence performance independently of the perceptual abilities being assessed. Controlling for loudness perception or sensation level at the individual level and testing under otherwise identical conditions may help reduce such confounding effects and could potentially affect the observed outcomes.

A strength of the present study is that the same individuals were assessed across multiple tasks, allowing different aspects of auditory processing and performance to be examined within the same participants. In addition, the CI and NH groups were matched on some characteristics. Nevertheless, the small sample size limits the conclusions that can be drawn from the findings. Future studies should include larger samples and, where possible, control for additional individual background factors. The CI group was heterogeneous regarding factors such as etiology of hearing loss, implant systems and devices, bilateral CI use or bimodal hearing, age at implantation, and bilingualism. Such heterogeneity is common within the CI population. Hearing outcomes following cochlear implantation are not always predictable and are influenced by numerous biological, demographic, and environmental factors. Surgical outcomes, anatomical conditions, comorbidities, device programming, daily device use, rehabilitation, and support from the home and school environment may all contribute to individual variation. Many of these factors are difficult to fully document and control for in research studies. Continued investigation of subgroups within the CI population and specific aspects of CI hearing is important.

Over time, implantation age has decreased, while CI technology, language-support interventions, and professional counseling for parents have continued to improve. As a result, spoken language outcomes and auditory development in children with CIs have generally improved, and language delays associated with hearing impairment have become less pronounced [55,56]. Since data collection for the present study was conducted several years ago, and the participants were implanted at somewhat older ages than is often seen today, the findings are specific to this cohort and cannot be assumed to generalize to more recently implanted populations without further investigation. Despite these limitations, the children with CIs in our study demonstrated the ability to detect brief acoustic changes, respond fast, and time responses well to brief auditory temporal cues. It is also possible that the participants represented a relatively high-achieving subgroup of children with CIs, choosing to participate on this type of test occasion during a summer camp with their family members and other peers with hearing impairment. Furthermore, previous experiences may have influenced performance and may partly explain the lack of group differences observed for some measures. In Sweden, children with CIs typically undergo regular clinical follow-ups, including repeated assessments of hearing, speech, and language development, whereas children with NH often have less experience with structured testing situations. This difference in test familiarity may have influenced task performance. In addition, a greater proportion of the children in the CI group reported playing a musical instrument compared with the NH group (Table 1). Previous research has shown that adult musicians with NH tend to exhibit lower GIN thresholds than non-musicians [57], suggesting that auditory temporal resolution may, at least partly, be influenced by auditory experience. Similarly, musically trained individuals have demonstrated enhanced sensorimotor timing precision during finger tapping tasks [58]. Therefore, musical experience represents a potentially relevant factor that was not accounted for in the present study and may have contributed to some of the observed differences between groups.

## 5. Conclusions

This study explored auditory temporal processing abilities across multiple levels and domains and provides insight into several aspects of auditory processing in children with CIs. However, the relationships among the different tasks, as well as the extent to which sensitivity to brief acoustic properties contributes to speech perception and language processing, require further investigation. These children’s CIs seem to provide the possibility to perceive short temporal changes in time, to react fast to simple acoustic stimuli, as well as to integrate temporal auditory information with good precision when tapping to a beat. Although the children with CIs in our test setup did not detect as short gaps in noise as the age-, sex-, and IQ-matched normal hearing children did, their cochlear devices nevertheless supported fast sound transmission with the ability to detect temporal changes in time below tens of milliseconds. In contrast, the nonword repetition task revealed challenges through CI hearing. Different consonant types resulted in varying levels of difficulty, with these effects being more pronounced in the CI group than in the normal hearing group. A potential pattern based on our overall findings is that the complexity of the acoustical input may be crucial for the CI performance. The level of performance approached those of the normal hearing listeners in tasks involving relatively simple auditory stimuli with no other competing input, such as reaction time and finger tapping, whereas larger differences emerged in tasks involving more complex acoustic or speech signals, including gap detection in noise and nonword repetition, while also containing rapid changes between sounds or noises. This potentially reflects limitations in the representation of the sound stream or speech signal through a CI more easily masked by other components. However, this hypothesis needs to be tested. Moreover, the distinct performance patterns observed across the different tests may suggest that temporal resolution, reaction time, sensorimotor synchronization, and phonological processing rely on partly distinct underlying mechanisms rather than a single, interchangeable temporal capacity.

To establish word recognition and develop language, a child must form phonological representation through perceiving changes in the speech signal and discriminate accurately between speech sounds. At least some children with CIs continue to exhibit challenges in establishing precise phonological representations, particularly under more demanding listening conditions. As a result, they may require greater exposure and training to learn and recognize novel sound patterns in the absence of prior contextual or semantic knowledge. Future studies involving larger cohorts and more extensive test batteries are needed to further explore the role of temporal auditory processing in CI hearing. Investigating subgroups of children implanted at younger ages may improve our understanding of how early auditory experience influences the development of temporal processing, phonological representations, and speech perception. Whether targeted auditory training focused on phonological processing or challenging listening situations can enhance temporal processing and ultimately improve speech perception in children with CIs remains an important question.

## Figures and Tables

**Table 2:** Group comparisons of nonword repetition (NWR) performance between the 15 children with normal hearing (NH) and the 15 children with cochlear implants (CI) using Kruskal–Wallis H tests (one-way ANOVA on ranks). The table presents test outcomes, effect sizes (epsilon-squared, *ε*^2^), mean ranks, and percentages of correctly repeated vowels, consonants, and complete nonwords, both overall and for each of the three subtests separately. In general, children in the NH group achieved higher scores than children in the CI group. The adjusted significance threshold following Bonferroni correction for multiple comparisons was <0.004.

	Total	Subtest I	Subtest II	Subtest III
**Complete nonwords**				
Kruskal–Wallis H	21.629	17.992	19.802	4.502
*p*-value (Asymp. Sig)	<0.001	<0.001	<0.001	0.034
Effect size, *ε*^2^	0.737	0.607	0.672	0.125
Mean % (*SD*) NH	61 (13)	84 (18)	72 (15)	26 (20)
Mean % (*SD*) CI	27 (13)	36 (22)	33 (14)	12 (14)
Mean rank NH	22.97	22.27	22.57	18.80
Mean rank CI	8.03	8.73	8.43	12.20
**Vowels**				
Kruskal–Wallis H	14.311	15.124	11.346	4.142
*p*-value (Asymp. Sig)	<0.001	<0.001	<0.001	0.042
Effect size, *ε*^2^	0.457	0.504	0.370	0.112
Mean % (*SD*) NH	94 (4)	96 (5)	97 (3)	90 (7)
Mean % (*SD*) CI	84 (10)	82 (12)	89 (7)	81 (14)
Mean rank NH	21.57	21.67	20.80	18.73
Mean rank CI	9.43	9.33	10.20	12.27
**Consonants**				
Kruskal–Wallis H	13.184	18.569	18.885	7.632
*p*-value (Asymp. Sig)	<0.001	<0.001	<0.001	0.006
Effect size, *ε*^2^	0.435	0.627	0.639	0.237
Mean % (*SD*) NH	88 (6)	98 (4)	94 (4)	76 (12)
Mean % (*SD*) CI	75 (10)	85 (7)	81 (11)	63 (14)
Mean rank NH	21.33	22.30	22.43	19.93
Mean rank CI	9.67	8.70	8.57	11.07

**Table 3:** Group comparisons of correctly repeated consonant types during nonword repetition (NWR) between the 15 children with normal hearing (NH) and the 15 children with cochlear implants (CIs) using Kruskal–Wallis H tests (one-way ANOVA on ranks). The table presents test outcomes, effect sizes (epsilon-squared, *ε*^2^), mean ranks, and percentages of correctly repeated fricatives, laterals (/L/), plosives, /r/ sounds, and nasals. Children in the NH group achieved significantly higher scores than children in the CI group for plosives, fricatives, /r/ sounds, and nasals, whereas no significant group difference was observed for laterals (/L/). Following Bonferroni correction for multiple comparisons, the adjusted significance threshold was <0.01.

	Fricatives	Laterals	Plosives	/r/	Nasals
**Consonant types**					
Kruskal–Wallis H	9.901	1.015	6.896	7.218	18.158
*p*-value (Asymp. Sig)	0.002	0.314	0.002	0.007	<0.001
Effect size, *ε*^2^	0.318	0.001	0.211	0.222	0.613
Mean % (*SD*) NH	93 (6)	90 (6)	87 (7)	90 (10)	85 (11)
Mean % (*SD*) CI	83 (10)	87 (8)	75 (12)	77 (13)	61 (10)
Mean rank NH	20.47	17.07	19.70	19.67	22.30
Mean rank CI	10.53	13.93	11.30	11.33	8.70

**Table 4:** Within-group comparisons of percentages of correctly repeated consonants during nonword repetition (NWR) across subtests and consonant types for the 15 children with normal hearing (NH) and the 15 children with cochlear implants (CIs). The table presents results from Friedman tests (*χ*^2^), with effect sizes reported as Kendall’s *W* (Bonferroni-adjusted significance threshold: <0.0125), followed by post hoc Wilcoxon signed-rank tests (*Z*) with effect sizes (*r*) (Bonferroni-adjusted significance threshold: <0.002). For both the NH and CI groups, significant differences between subtests were driven by lower consonant scores in the third subtest compared with the first two subtests. This pattern was observed when consonant clusters that do not occur in Swedish were introduced in the third subtest. Significant differences between consonant types were observed only within the CI group, indicating that some consonant types were more difficult to repeat correctly than others.

	NH Group			CI Group		
**Friedman test**	*χ* ^2^	*W*	*p*-value	*χ* ^2^	*W*	*p*-value
Subtests	26.069	0.87	<0.001	20.800	0.69	<0.001
Consonant types	18.593	0.31	<0.001	41.333	0.69	<0.001
**Wilcoxon signed-rank test**	*Z*	*r*	*p*-value	*Z*	*r*	*p*-value
Subtest I and II	−2.205	0.57	0.027	−2.188	0.57	0.029
Subtest I and III	−3.411	0.88	<0.001	−3.408	0.88	<0.001
Subtest II and III	−3.411	0.88	<0.001	−3.294	0.85	<0.001
Fricatives and Laterals	−2.170	0.56	0.030	−2.136	0.55	0.033
Fricatives and Plosives	−2.957	0.76	0.003	−3.238	0.84	0.001
Fricatives and /r/	−1.125	0.29	0.260	−1.537	0.40	0.124
Fricatives and Nasals	−2.732	0.71	0.006	−3.410	0.88	<0.001
Laterals and Plosives	−2.216	0.57	0.027	−3.408	0.88	<0.001
Laterals and /r/	−0.283	0.07	0.777	−3.126	0.81	0.002
Laterals and Nasals	−2.315	0.60	0.021	−3.413	0.88	<0.001
Plosives and /r/	−1.648	0.43	0.099	−0.596	0.15	0.551
Plosives and Nasals	−0.909	0.24	0.363	−3.124	0.81	0.002
/r/ and Nasals	−2.263	0.58	0.024	−3.186	0.82	0.001

## Data Availability

The data presented in this study are available upon request from the corresponding author. The dataset is not publicly available due to ethical reasons. Participants and their caregivers provided consent for the publication and presentation of aggregate study results only. As the complete dataset contains detailed participant-level information and the population of children with cochlear implants in Sweden is relatively small, there is a risk of participant re-identification.

## Abbreviations

The following abbreviations are used in this manuscript:

CI(s)	Cochlear Implant(s)
NH	Normal Hearing
GIN	Gap detection In Noise
RT(s)	Reaction Time(s)
NWR	Nonword Repetition
IRI(s)	Inter-Response Interval(s)
